# Complete Genome Sequence of the First Colistin-Resistant Raoultella electrica Strain

**DOI:** 10.1128/mra.00047-23

**Published:** 2023-04-04

**Authors:** Claudia Aldeia, Edgar I. Campos-Madueno, Parham Sendi, Andrea Endimiani

**Affiliations:** a Institute for Infectious Diseases, University of Bern, Bern, Switzerland; b Graduate School of Cellular and Biomedical Sciences, University of Bern, Bern, Switzerland; University of Maryland School of Medicine

## Abstract

We present the full genome sequence of a colistin-resistant Raoultella electrica strain (MIC, >4 μg/mL) that was isolated from the stool of a healthy person living in India. The sequence consists of a chromosome and three plasmids (5,455,992-bp and 98,913-bp, 4,232-bp, and 3,961-bp, respectively). No previously described colistin resistance mechanisms were detected.

## ANNOUNCEMENT

*Raoultella* spp. are ubiquitous members of the family *Enterobacteriaceae* (1, 2). Specifically, Raoultella electrica has been reported in animals and the environment but not in humans (3, 4). Only one complete genome sequence of R. electrica is available (GenBank accession number CP041247.1), a strain (DSM 102253^T^) isolated from a fuel cell (4, 5). Moreover, no evidence of colistin resistance in this species was reported previously (3, 4). Here, we report the complete genome sequence of R. electrica strain S2-IND-01-C, which was isolated in 2022 from the stool of a healthy man living in India. The investigation is part of a cohort study (https://data.snf.ch/grants/grant/192514) that was approved by the ethics committee of the Canton Bern, Switzerland (ID 2020-01683).

A fresh stool sample (~50 μg) was screened for colistin-resistant *Enterobacterales* strains by preenrichment for 6 h in Luria-Bertani broth supplemented with colistin (1 μg/mL), followed by overnight plating (100 μl) on CHROMID Colistin R agar (bioMérieux) at 37°C. Colonies were subcultivated overnight on MacConkey II agar (Becton-Dickinson) at 37°C. Species identification with a matrix-assisted laser desorption ionization–time of flight mass spectrometry (MALDI-TOF MS) system (Bruker) indicated that the closest match was Raoultella ornithinolytica. Susceptibility testing using the GNX2F Sensititre panel (Thermo Fisher Scientific) determined that S2-IND-01-C was resistant to colistin (MIC, >4 μg/mL) (Table 1).

**TABLE 1:** Antimicrobial susceptibility profile for R. electrica strain S2-IND-01-C

Antibiotic(s)	MIC (μg/mL)^*a*^
Piperacillin-tazobactam	≤8/4 (S)
Ticarcillin-clavulanate	≤16/2 (S)
Ceftazidime	≤1 (S)
Cefotaxime	≤1 (S)
Cefepime	≤1 (S)
Aztreonam	≤2 (S)
Imipenem	≤1 (S)
Meropenem	≤1 (S)
Doripenem	≤0.12 (S)
Ertapenem	≤0.25 (S)
Gentamicin	≤1 (S)
Tobramycin	≤1 (S)
Amikacin	≤4 (S)
Ciprofloxacin	≤0.25 (S)
Levofloxacin	≤1 (S)
Colistin	>4 (R)^*b*^
Polymyxin B	>4 (NA)^*b*^
Doxycycline	≤2 (NA)
Minocycline	≤2 (NA)
Tigecycline	≤0.25 (S)
Trimethoprim-sulfamethoxazole	≤0.5/9.5 (S)

a Antimicrobial susceptibility was determined using the Sensititre GNX2F panel. MICs were interpreted according to the 2022 European Committee on Antimicrobial Susceptibility Testing (EUCAST) criteria for *Enterobacterales* (https://www.eucast.org/fileadmin/src/media/PDFs/EUCAST_files/Breakpoint_tables/v_12.0_Breakpoint_Tables.xlsx). R, resistant; S, susceptible; NA, interpretative criteria not available.

b Nonsusceptibility to polymyxins.

Using the PureLink microbiome DNA Purification Kit (Thermo Fisher Scientific), DNA was isolated from a reactivated bacterial glycerol stock that had been subcultivated overnight on MacConkey II agar. A short-read whole-genome sequencing (WGS) library was prepared with the NEBNext Ultra II DNA library preparation kit and sequenced on a NovaSeq 6000 instrument (2 × 150-bp paired-end reads; Illumina). For long-read WGS, the library was prepared using the SQK-RBK004 rapid barcoding kit, loaded on a FLO-MIN106D R9 flow cell, and analyzed for 48 h on a MinION sequencer (Oxford Nanopore Technologies [ONT]). Long-read base calling was performed with Guppy v3.4.5 (ONT). Long reads were adapter trimmed and quality filtered with Porechop v0.2.4 and Filtlong v0.2.1 (minimum length, 1,000-bp; number of target bases, 1 billion), whereas short reads were preprocessed with Trimmomatic v0.36 (6, 7). Unless specified otherwise, all bioinformatic steps were performed with default parameters.

Sequencing generated 8,487,292 short reads and 436,545 long reads (*N*_50_, 11,250-bp). A complete and circular *de novo* assembly with an average sequencing depth of 425× and a GC content of 49.9% was generated using the hybrid pipeline from Unicycler v0.4.8 (8, 9). The genome consisted of a chromosome (5,455,992-bp) and three plasmids, i.e., p1-S2-IND-01-C (98,913-bp), p2-S2-IND-01-C (4,232-bp), and p3-S2-IND-01-C (3,961-bp). The chromosome and p1-S2-IND-01-C were rotated by Unicycler to *dnaA* and *repA*, respectively.

TYGS and JSpeciesWS analysis indicated that S2-IND-01-C actually belonged to the species Raoultella electrica, with 98.74% average nucleotide identity with the reference strain DSM 102253^T^ (10, 11). The NCBI Prokaryotic Genome Annotation Pipeline (PGAP) identified 5,384 genes (5,265 coding sequences and 119 RNA genes). The presence of antimicrobial resistance genes (ARGs) and plasmid replicon sequences was assessed using the Comprehensive Antibiotic Resistance Gene Identifier v6.0.0 and the Center for Genomic Epidemiology web tools ResFinder v4.1 and PlasmidFinder v2.0.1 (50% identity and 60% coverage) (12–14). S2-IND-01-C carried chromosomal *bla*_PLA_-, *fosA*-, *qacL*-, *oqxA*-, and *oqxB*-like ARGs (91.4%, 95.2%, 92.2%, 84.0%, and 87.7% identity, respectively). Plasmids p1-S2-IND-01-C, p2-S2-IND-01-C, and p3-S2-IND-01-C were not associated with ARGs and possessed IncFII(Yp), Col440I, and Col440I replicon sequences, respectively.

Despite the colistin-resistant phenotype, mobile colistin resistance (*mcr*)-like genes were not found in S2-IND-01-C. Furthermore, comparisons with DSM 102253^T^ showed identical amino acid sequences for MgrB, PmrA/B, and PhoP/Q (chromosomal regulators of the lipopolysaccharide [LPS] modifications) (15). Therefore, R. electrica may be naturally resistant to colistin. However, other strains belonging to this species should be phenotypically and genotypically tested to determine the underlying mechanism of resistance.

### Data availability.

The genome of R. electrica S2-IND-01-C has been deposited in GenBank under accession numbers CP112887.1, CP112888.1, CP112889.1, and CP112890.1. Sequencing reads are available in the Sequence Read Archive (SRA) under SRA accession numbers SRR23117923 and SRR23117922 for Illumina and Nanopore reads, respectively.
